# Habitat Selection and Home Range of Reeves’ Turtle (*Mauremys reevesii*) in Qichun County, Hubei Province, China

**DOI:** 10.3390/ani13091514

**Published:** 2023-04-30

**Authors:** Rongping Bu, Zihao Ye, Haitao Shi

**Affiliations:** Ministry of Education Key Laboratory for Ecology of Tropical Islands, College of Life Sciences, Hainan Normal University, Haikou 571158, China

**Keywords:** habitat selection, home range, endangered species, overexploitation, turtle conservation

## Abstract

**Simple Summary:**

This study investigated Reeves’ turtle (*Mauremys reevesii*) using radiotelemetry to determine its habitat selection and home range in Qichun County, Hubei Province, China. The results show that this is a semi-aquatic species and not purely aquatic, as previously categorized. When active in terrestrial habitats, these turtles prefer low-canopy cover habitats near the edges of fields with abundant shelter. They also prefer areas with many shelter opportunities in aquatic habitats, particularly shallow habitats near the water’s edge. Our study’s average home range area was larger than those noted in previous studies. Furthermore, many human activities occurred within their active area, threatening the Reeves’ turtle population. Protecting the habitat of Reeves’ turtles is crucial for their survival, and efforts should be made to preserve some of their original habitat from exploitation. Our results fill a gap in knowledge of the natural history of this endangered species.

**Abstract:**

Habitat selection and range are crucial factors in understanding the life history of species. We tracked 23 adult wild Reeves’ turtles (*Mauremys reevesii*) from August 2021 to August 2022 in Qichun County, Hubei Province, China, to study their habitat selection, home range, and the characteristics of chosen habitats. Significant differences were observed in aquatic habitats, regarding shelter cover (*Z* = −6.032, *p* < 0.001), shelter height (*Z* = −6.783, *p* < 0.001), depth of water (*Z* = −2.009, *p* = 0.045), and distance from the edge (*Z* = −4.288, *p* < 0.001), between selected and random habitats. In terrestrial habitats, significant differences were observed in canopy cover (*Z* = −2.100, *p* = 0.036), herbage cover (*Z* = −2.347, *p* = 0.019), distance from the field edge (*Z* = −2.724, *p* = 0.006), dead grass cover (*Z* = −2.921, *p* = 0.003), and dead grass thickness (*t* = 3.735, *df* = 17, *p* = 0.002) between the selected and random habitats. The mean home range area observed for this turtle population was 14.34 ± 4.29 ha, the mean core home range was 2.91 ± 2.28 ha, and the mean line home range was 670.23 ± 119.62 m. This study provides valuable information on this endangered species, providing a foundation for the development of conservation plans.

## 1. Introduction

Wildlife is an important ecosystem component and is vital in maintaining the ecological environment on which human beings depend for survival [1]. Wild vertebrates face multiple threats and challenges. Currently, wild vertebrate populations are rapidly declining, and approximately 20% of species face a risk of extinction [2,3]. Human activities are some of the most significant drivers of vertebrate extinction risks globally [4]. Turtles, in particular, with their high economic, cultural, and ornamental value, are popular targets for hunting and are subsequently kept as pets, used in traditional Chinese medicine, or consumed as food [5,6]. Consequently, turtles are the most threatened group of vertebrates, with more than half of all species at risk of extinction [6]. Despite their vulnerable status, the ecology of Asian turtles remains poorly understood due to the scarcity of populations, making it challenging to conduct targeted ecological studies [7]. Improving our understanding of the ecology of wild turtles is fundamental for effective conservation efforts [8].

Habitat destruction and degradation are among the primary factors contributing to the dramatic decline in turtle populations [9,10]. Habitat is essential for animal survival and reproduction, providing food, shelter, and an environment for competition, predation, and mating [11,12,13]. Home range refers to the sum of the areas used and traversed by individual animals or groups to complete their life activities [14]. Therefore, habitat selection and home range studies play a vital role in identifying critical habitats, evaluating habitat quality, and understanding the spatial ecology of a species [8,15,16]. These are of great significance for effective habitat planning and management, as well as the conservation of endangered species [17,18,19].

**Figure 1:**
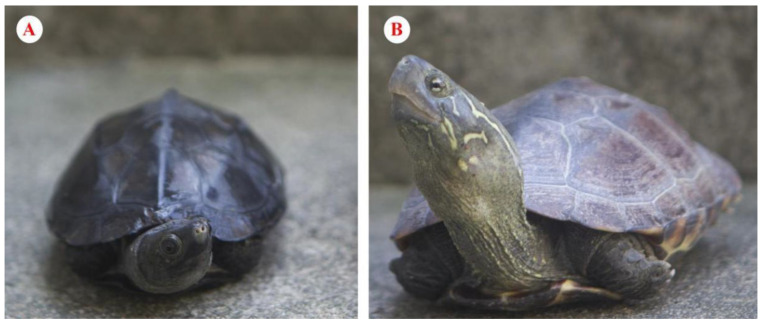
Male (**A**) and female (**B**) adult *Mauremys reevesii* show distinct sexual dimorphism in size and body color.

The Reeves’ turtle (*Mauremys reevesii*) (Figure 1) is a species widely distributed in East Asia throughout central and eastern continental China, southern Japan, and part of the Korean peninsula [20]. However, this endangered species requires urgent conservation efforts because individuals have been regularly captured over a long period, and their habitats are being destroyed [20,21,22]. Consequently, its population has plummeted, leading to its classification as an endangered species by the International Union for Conservation of Nature [23]. Furthermore, due to their low numbers in China, identifying a suitable study population of the Reeves’ turtle has become difficult, impeding field ecological investigations [7]. Therefore, fundamental ecological knowledge, including habitat utilization characteristics, quality, and range, is lacking, impeding an understanding of the environmental requirements of wild turtles in their natural habitat and hindering efforts to evaluate and protect this species.

The Reeves’ turtle has long been an enigmatic species, and ecological studies on this animal are lacking. Lovich et al. [20] thoroughly characterized this species and categorized it as an aquatic species, principally on the basis of preliminary observations in Japan [24,25,26,27]. However, Song et al. [28] indicated that this species inhabits orchards and woodlands in South Korea, and Haramura et al. [29] demonstrated the extensive use of terrestrial habitats by this turtle in Japan. Home range studies of the Reeves’ turtles have been limited, with only a few conducted in introduced populations. A study in Korea found the maximum seasonal travel distance of Reeves’ turtle to be 196 m, with a home range of 2.6–8.1 ha [28]. Haramura et al. [29] conducted another study in Japan that showed a nesting season home range of 1–60 ha, with a maximum daily movement of approximately 500 m. However, notably, different populations of the same turtle species can exhibit significantly different home ranges [30].

In China, where the Reeves’ turtle was first recorded, and where the largest natural population is found historically, there has been no study on its habitat or home range, posing a barrier to assessing the species. We have long wanted to conduct field ecological research on this species in China but could not do so because we could not find a suitable field population. However, in 2021, we found a wild population of this species in Qichun County, Hubei Province. Turtles have long been used in traditional Chinese medicine in Qichun County [31], and the wild population has been under serious capture pressure for a long time [22]. Therefore, our study of habitat selection and home range for the wild Reeves’ turtle in Qichun County is important for assessing the habitat status of its wild population and essential to conserving this species.

Our study describes Reeves’ turtle home ranges and habitat selection and reveals how these parameters affect Reeves’ turtles under original habitat and population conditions, filling a gap in knowledge of the natural history of this endangered species.

## 2. Materials and Methods

### 2.1. Habitat Selection

**Figure 2:**
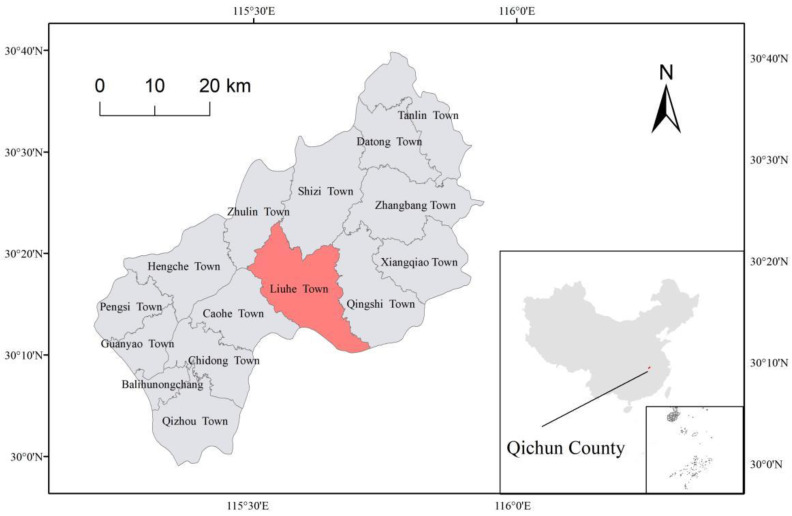
Map of the study area. The red area on the big map indicates Liuhe Town (the field tracking area), and the red area on the small map indicates the location of Qichun County.

The research was conducted in Qichun County (29°59–30°40′ N, 115°12′–115°56′ E) (Figure 2), located in Hubei Province, China. Qichun County is characterized by a subtropical continental monsoon climate with four seasons, a mild climate, sufficient light, and abundant rainfall. The average annual temperature is 16.8 °C, with summer extremes as high as 39.7 °C ranging from mid-May to mid-September and winter extremes as low as −15.6 °C ranging from mid-December to mid-February. The average annual relative humidity is 80%, with the highest being 82% in February–March and the lowest being 77% in August. The study area is hilly, and the vegetation mainly comprises secondary forests [32].

**Figure 3:**
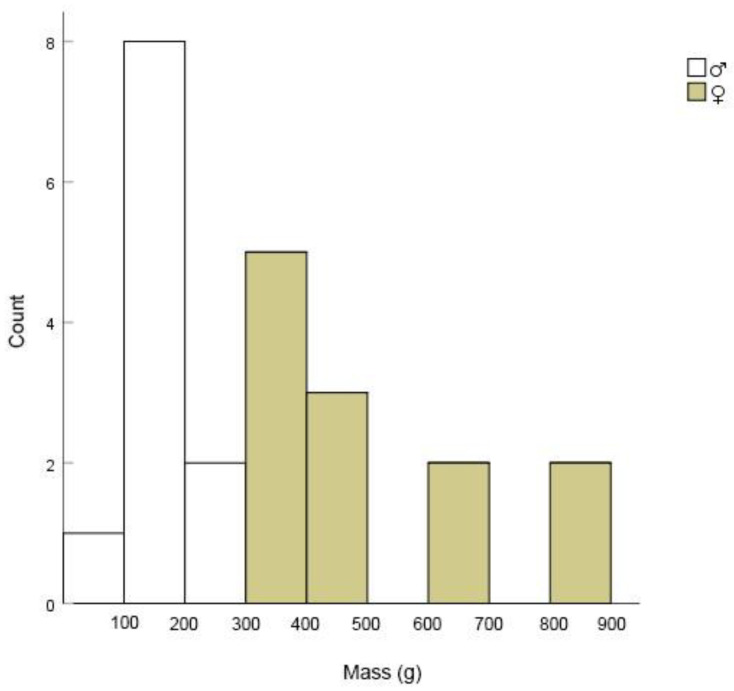
Mass–class distributions of Reeves’ turtles (*Mauremys reevesii*) tracked during field surveys in Qichun County, Hubei Province, China.

**Figure 4:**
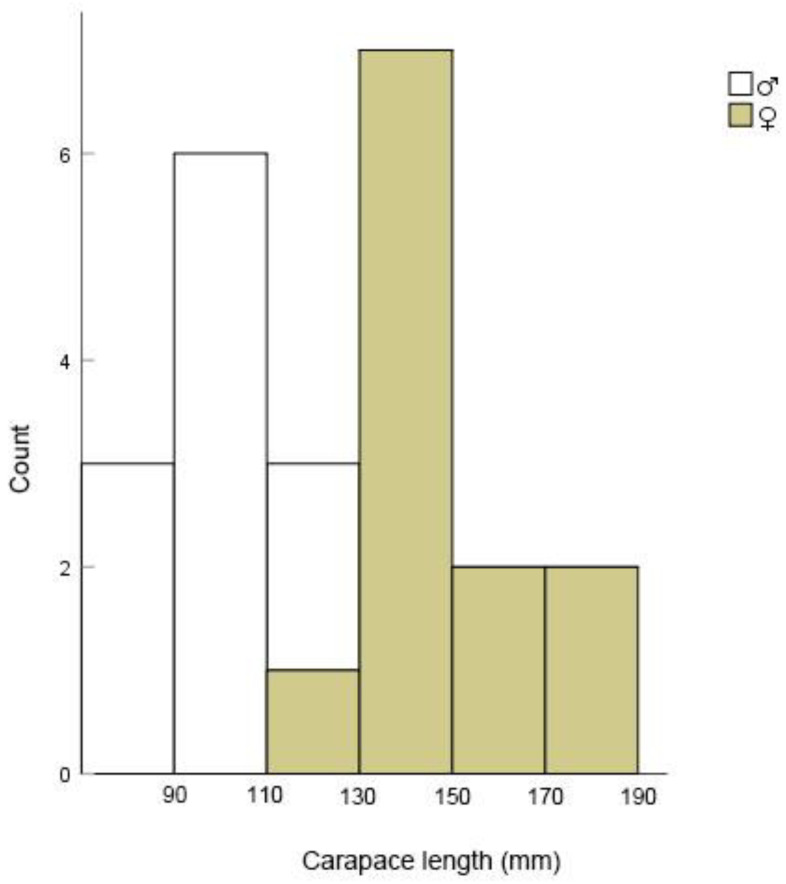
Carapace length–class distributions of Reeves’ turtles (*Mauremys reevesii*) tracked during field surveys in Qichun County, Hubei Province, China.

**Figure 5:**
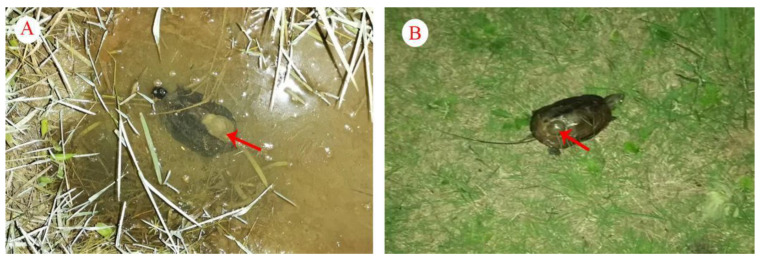
Turtles with transmitters in their natural habitat, where (**A**) is a male in an aquatic habitat, and (**B**) is a female in a terrestrial habitat. The red arrow indicates the transmitter.

Using radio telemetry, the habitat selection and home range of 23 wild Reeves’ turtles (11 males and 12 females) (Figure 3 and Figure 4) were studied from August 2021 to August 2022. These turtles were caught by placing nylon cages (length 300 cm, cross-section 30 × 20 cm) in natural ponds. All individuals were brought back to the study base, measuring the carapace length with vernier calipers (Shanghai Tool Works Co. Ltd., Shanghai, China), and weighing the mass with an electronic balance (Shanghai Hochoice Apparatus Manufacturer Co., Ltd., Shanghai, China). Females with carapace lengths >110 mm were regarded as adults, and males with mean carapace lengths >87 mm were regarded as adults [22]. Each adult turtle was equipped with a radio tracking transmitter (RI-2B, 216.000–216.999 MHz; Holohil Systems, Ltd., Ottawa, ON, Canada) at the posterior terminus of the carapace, using a mixture of epoxy resin and epoxy epoxide hardener (Figure 5). The turtle’s weight and size determined the size of the transmitter, with an 8 g transmitter used for 15 individuals and a 6 g transmitter used for eight small male individuals to ensure that the transmitter weight did not exceed 8% of the turtle’s mass. All transmitters were placed at the posterior end of the carapace to avoid any hindrance to microhabitat usage. The turtles were released at the same location where more than two individuals were collected from the wild, and a handheld receiver (TRX-1000S, 216.000–216.999 MHz; Wildlife Materials International, Inc., Murphysboro, IL, USA) with a three-component folding antenna was used to locate each turtle daily. After the tracking study, the turtles were recaptured, and the transmitters were removed from their carapaces.

The study identified selection sites as locations where each turtle appeared at least five times or stayed for more than 3 days. For each selected site, data were gathered on the type of habitat (aquatic or terrestrial), distance from human settlements with more than two households, and distance from human disturbances such as grazing, agriculture, fish feeding, and human capture. Two quadrat sizes (10 × 10 m and 1 × 1 m) were used to analyze the ecological factors within the selected sites. For each selected site, a random quadrat was also chosen within a range of 10–50 m in a random direction, which is positioned by a random function in Microsoft Excel. The minimum separation of 10 m ensured no overlap between the random and selected quadrats, while the maximum separation of 50 m was based on the average home range length of a Reeves’ turtle [28]. If the random site had the same habitat characteristics as the selected site, it was recorded as the random habitat. However, if the habitat types differed, the random site was discarded and randomly repositioned until the habitat types matched. In the larger terrestrial quadrats, we measured the canopy cover (%) and vegetation cover (%), whereas, in the smaller terrestrial quadrats, we analyzed the canopy cover (%), slope gradient (°), herbage cover (%), herbage height (cm), leaf litter cover (%), leaf litter thickness (thickness of fallen leaves from surface to ground, cm), distance from water (m), and distance from the field edge (m). Similarly, in the larger aquatic quadrats, we quantified the canopy cover (%), vegetation (vegetation growing over the water) cover (%), and pH, whereas, in the smaller aquatic quadrats, we examined the canopy cover (%), shelter cover the proportion of the surface shelter (e.g., herbage, branches), %), shelter height (cm), distance from shore (edge) (m), depth of water (cm), and water flow velocity (cm/s).

### 2.2. Home Range

Each individual turtle was located once per day, and its latitude and longitude were recorded by a handheld Hengyili latitude and longitude measuring instrument S7 (Hengyili Technology Co. Ltd., Chengdu, China). After collecting location data, a home range analysis was conducted using Home Range Tools for ArcGIS (Center for Northern Forest Ecosystem Research, Thunder Bay, ON, Canada) in ArcGIS 10.8 (Environmental Systems Research Institute, Inc., Redlands, CA, USA). The 95% minimum convex polygon (MCP) [33,34] thus estimated was used to determine the overall home range area. A 50% fixed kernel density estimation (FKDE) [35] was used to determine the core home range and the line home range, which is the straight distance between the two farthest locations [36,37]. The MCP method is the most widely used and conservative home range estimation method [38], and the calculated results of this method are well comparable to those of the home range study and are easy to calculate. The FKDE method is the best method to estimate home domain size and home domain utilization distribution. Moreover, it is one method that accurately estimates the home range area with the smallest deviation [39]. Line home range can reflect the activity intensity and activity ability of turtles over a period of time [37].

### 2.3. Statistical Analysis

The statistical analyses were conducted using SPSS Statistics for Windows, version 18.0 (SPSS Inc., Chicago, IL, USA). Prior to analysis, the data were evaluated for normality using the Kolmogorov–Smirnov test. For normally distributed data, paired t-tests were employed to compare the differences in environmental variables between the selected and random habitats, while non-normally distributed data were assessed using Wilcoxon signed-rank tests. Independent sample t-tests were utilized to determine the differences between males and females when the data met the normal distribution. Mann–Whitney U tests were utilized when the data were not normally distributed. The data were reported as the mean ± standard error (SE). Additionally, discriminant function analysis was employed to evaluate the discrepancies in the numeric variables between the selected and random habitats and to identify the variables that best separated them. The acceptance level was established at *p* < 0.05.

## 3. Results

### 3.1. Habitat Selection

Reeves’ turtles use terrestrial and aquatic habitats, with a ratio of approximately 4:1 (74:18) between aquatic and terrestrial habitats. In addition, they extensively use terrestrial habitats for activity and migration, comprising approximately 34% (467/1387) of their temporary active sites. The mean distance between selected habitats and human settlements (186.76 ± 0.54 m, 18–274 m) was significantly greater than that between selected habitats and human disturbance (62.74 ± 0.52 m, 0–150 m), indicating the turtles’ avoidance of human settlements. However, they could not avoid the unpredictable human disturbances. In aquatic habitats, the maximum water area was 1254 m^2^, with 72 selected habitats located in standing water and only two selected habitats in slow-moving streams. Reeves’ turtles demonstrated a strong preference for standing water, with approximately 90% (826/920) of their aquatic temporary active sites located in these areas.

Within the aquatic habitats, there were significant differences in vegetation cover (*Z =* −3.430, *p =* 0.001) between selected and random aquatic habitats at a broad scale (10 × 10 m). However, at a fine scale (1 × 1 m), there were significant differences in the shelter cover (*Z =* −6.032, *p* < 0.001), shelter height (*Z =* −6.783, *p* < 0.001), depth of water (*Z =* −2.009, *p =* 0.045), and distance from the edge (*Z =* −4.288, *p* < 0.001) (Table 1). This suggests that the turtles prefer low-canopy cover and shallow habitats near the water’s edge, which shelter them during their activities in aquatic habitats.

Regarding the terrestrial habitats, there was no significant difference in canopy and herbage cover at a broad scale (10 × 10 m) between the selected and random habitats. However, at a fine scale (1 × 1 m), there were significant differences in the canopy cover (*Z =* −2.100, *p =* 0.036), herbage cover (*Z =* −2.347, *p =* 0.019), distance from the field edge (*Z =* −2.724, *p =* 0.006), dead grass cover (*Z =* −2.921, *p =* 0.003), and dead grass thickness (*t* = 3.735, *df* = 17, *p =* 0.002) between the selected and random quadrats (Table 1). This indicates that Reeves’ turtles prefer low-canopy habitats near the field’s edges, which shelter them during their activities in terrestrial habitats.

When comparing males and females, only the pH difference was significant (*t* = −3.242, *df* = 72, *p* = 0.002) in aquatic habitats, with no significant difference observed in other environmental variables (Table 2). In contrast, for the difference between males and females in terrestrial habitats, the canopy cover of males was significantly higher than that of females at both broad (*t* = −3.165, *df* = 16, *p* = 0.006) and fine scales (*t* = −4.312, *df* = 16, *p* = 0.001), but there was no significant difference in other environmental variables (Table 2).

At a fine scale of 1 × 1 m, a comparison was made between selected and random aquatic and terrestrial habitats. The results of a stepwise discriminant analysis showed that shelter cover, shelter height, distance from the edge, and depth of water were significant factors in distinguishing the selected and random aquatic habitats, while the leaf litter thickness and dead grass thickness were significant in distinguishing the selected and random terrestrial habitats. The eigenvalue of the stepwise discriminant function was 1.064 for terrestrial habitats and 0.745 for aquatic habitats. In addition, the canonical correlation coefficients of terrestrial and aquatic habitats were 0.718 and 0.653, respectively. The stepwise discriminant functions for terrestrial and aquatic habitats explained 100% of the total variance. Wilks’ λ also showed a significant difference between the selected and random habitats for aquatic (Wilks’ λ = 0.485, *χ*^2^ = 104.315, *df* = 4, *p* < 0.001) and terrestrial (Wilks’ λ = 0.573, *χ*^2^ = 18.367, *df* = 2, *p* < 0.001) habitats. The correct discrimination rate was 83.8% for aquatic habitats and 80.6% for terrestrial habitats (Table 3).

### 3.2. Home Range

A total of 1387 locations of 23 Reeves’ turtles were identified, of which 79 locations were excluded in five individuals because their signals disappeared within a short period of time. After excluding lost individuals, the home ranges of 18 individuals (11 females and seven males) were analyzed. The analyses revealed no significant differences in home range area (F: 15.53 ± 4.99, M: 12.48 ± 8.25; *t* = 0.337, *df* = 16, *p* = 0.740), core home range area (F: 4.46 ± 3.72, M: 0.48 ± 0.18; *Z* = −0.589, *p* = 0.556), and line home range (F: 738.06 ± 140.88, M: 563.62 ± 221.74; *t* = 0.700, *df* = 16, *p* = 0.494) between the sexes. Thus, we pooled the home range data of male and female individuals for analysis.

The Reeves’ turtles’ mean home range area was 14.34 ± 4.29 ha (0.07–61.04 ha), the mean core home range area was 2.91 ± 2.28 ha (0.04–41.54 ha), and the mean line home range was 670.23 ± 119.62 m (68.75–1783.32 m). For the home range in different seasons, because there were fewer locations, and because the home range area error of the analyst was large, the line home range analysis was conducted for the positions in different seasons. In addition, one of the males died at the end of June (drowned in a cage placed by human), and the home range data for July and August were missing; therefore, the data of this individual in autumn were excluded when the mean line home range analysis was carried out. In the four seasons, the line home range of female turtles was significantly larger than that of male turtles only in summer (*t* = 2.838, *df* = 15, *p* = 0.012), and there was no significant difference in other seasons (Table 4).

**Figure 6:**
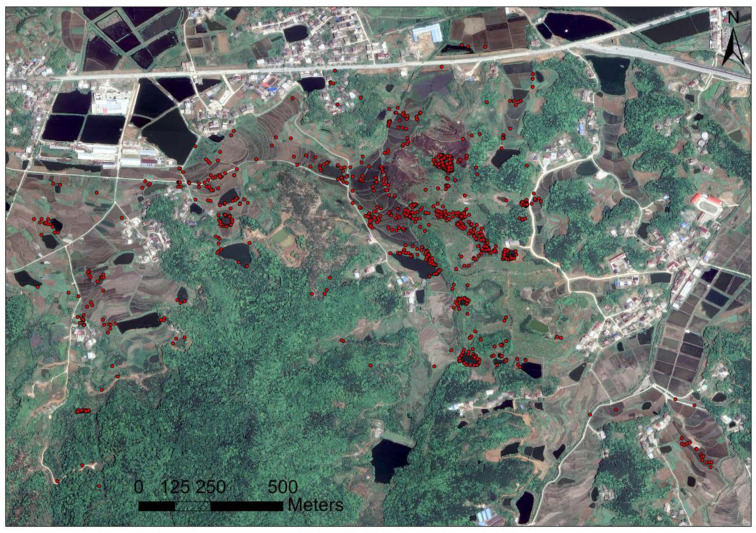
The locations within the active area of the Reeves’ turtles in Qichun County, Hubei Province. The red points indicate their locations.

All the locations on the map were marked in ArcGIS 10.8 to clarify the distribution within the home range. In the active area of these turtles, there were numerous villages and extensive areas of farmland, in which they have been active for a long time (Figure 6).

## 4. Discussion

The Reeves’ turtle has historically been classified as an aquatic species [16]. However, our tracking study revealed that, in addition to aquatic habitats, these turtles also utilize terrestrial habitats, including waste ground (land that was once farmed but is now abandoned), bamboo forests, and orchards. Moreover, we observed that several temporary active sites are located in terrestrial habitats, and migration through these habitats is frequent during the active period (from April to November). Our earlier study also revealed that turtles use terrestrial habitats for hibernation [40]. Previous studies conducted in South Korea and Japan also documented Reeves’ turtles’ use of terrestrial habitats [28,29]. Thus, we suggest that the species be classified as semi-aquatic instead of purely aquatic, as it has traditionally been categorized.

The habitat of Reeves’ turtle is characterized by a low canopy cover, which is thought to facilitate thermoregulation by the turtles [41,42]. Aquatic habitats preferred by the turtles are shallower areas near the water’s edge where shelter is abundant, such as ponds, rice paddies, abandoned farmland, and marshes. Preference for such shoreline habitats may be related to higher water temperatures [43]. Furthermore, the turtles’ aquatic temporary active sites are primarily in standing water. Among the 74 identified aquatic habitats, 72 were located in standing water, with only two in slow-moving streams. Standing water is preferred because it provides easier access to food and shelter than fast-flowing water [44]. In addition, the shallow water areas provide a high degree of shelter and are likely to be selected by foraging turtles for the greater food abundance, such as plant material, insect larvae, crustaceans, mollusks, amphibians, and fish, compared to those encountered in deep open water. Furthermore, the shelter can be a hiding place in an emergency [44,45]. Usuda et al. [46] found that changes in rivers within their range would affect the distribution, density, and abundance of Reeves’ turtles, but the population in our study rarely used river habitats, which consequently may have impacted them little.

In terrestrial habitats, the turtles prefer low-canopy cover habitats near the edges of fields, providing many shelter opportunities. The choice of open habitat is consonant with the turtles’ needs for basking, nesting, foraging, and overwintering [47]. The canopy cover of females was significantly lower than that of males when selecting terrestrial habitats. Melanin plays an important role in regulating body temperature, dark carapace helps with heating, and dark individuals have lower skin reflectivity, requiring less light to raise the same temperature [48]. Male Reeves’ turtles are darker than females and have an advantage in thermoregulation. Females select lower canopy cover habitats that receive more sunlight to raise the temperature. In addition, open habitats are subject to less predation pressure, as wild boars are abundant in woodlands in the study area, and there may have been more large predators historically. However, these open habitats may increase their risk of death when exposed to farming activities [49]. Overgrazing in turtle habitats can damage soil structure and vegetation, reducing the suitability of the habitat [50]. Conversely, the vegetation at the edges of fields is generally better preserved and effectively shelters the turtles. Selecting habitats with more herbage provides the turtles with additional shelter, consequently reducing the risk of predation, as well as providing safe places to bask and hibernate [51].

The average home range area of the individuals analyzed in our study was larger than that in a previous study in South Korea [28]. This difference can be attributed to the fact that we sampled each turtle relatively more intensively, over a longer period, allowing them to move around a wider range. Alternatively, it could be because of differences in the studied populations [8,30,52]. The largest home range area was similar to that reported in Japan [29], even though we tracked the turtles for extended periods. The fact that the home range of Reeves’ turtle is significantly smaller than that of aquatic turtles (41.29 ha) but is closer to semi-aquatic species (12.08 ha) and terrestrial species (14.21 ha) [30] suggests that this species is not exclusively aquatic. The mean line home range is larger when the temperature is higher, with the largest in summer and the second largest in autumn. In addition, the line home range of females in summer is larger than that of males, mainly because females lay eggs in summer and need to move a wider range to find suitable nesting sites (Z. Ye, unpublished data).

Within the active area of Reeves’ turtles, a substantial portion is occupied by human settlements, posing a significant threat to their survival. During our tracking period, two individuals entered the human settlements, after which the signal could not be traced, and the villagers reported that they had been captured and sold. Three others were caught in cages in ponds near human settlements. Two of them were taken away, and one drowned in a cage. Previous studies support this possibility, as studies have found that the population of yellow-spotted river turtles (*Podocnemis unifilis*) and red-eared sliders (*Trachemys scripta*) declined significantly in areas near human settlements [53,54]. Furthermore, the home range of Reeves’ turtles also contains many agricultural modifications, including rice paddies and temporary waste ground; these can provide turtles with shelter and food, but farming activities can pose a risk to their survival [55]. Because of their poor mobility [56], they cannot quickly avoid sudden agricultural harvest, exposing them to bare farmland and increasing their risk of capture. Of the individuals we tracked, three individuals were exposed during the rice harvest; one was taken away, and the other two were returned to us for further tracking. Although rice paddies have been considered areas that provide a rich food source for the turtles [57], the use of fertilizers and pesticides in farming can lead to water pollution and poison the insects that the turtles consume [58]. Moreover, many roads between the different water bodies constrain the turtles’ movement, and they only migrate at night when there is less human activity, such as motorized traffic and pedestrians (R. Bu, unpublished data). Therefore, they restrict easy movement between patches of suitable habitat and increase opportunities for accidental mortality [59,60,61]. Considering the amount of human encroachment into their habitat and the fact that turtles were captured during the study, this stress level can possibly lead to the rapid extinction of the population [62,63].

## 5. Conclusions

In summary, the Reeves’ turtle should be classified as semi-aquatic rather than purely aquatic as it has traditionally been categorized. When active in terrestrial habitats, the turtles prefer low-canopy cover habitats near the edges of fields where shelter is abundant, while, in aquatic habitats, they prefer shallow habitats near the water’s edge where shelter is abundant. Our study’s mean home range area was larger than that observed in previous studies, and there were numerous human disturbances in the active area, which threatened the Reeves’ turtle population. Protecting the habitat of Reeves’ turtles is crucial for their survival, and efforts should be made to preserve some of their original habitat from exploitation. Despite our ability to observe the immediate impacts of human activities on the Reeves’ turtle populations, there is a dearth of long-term population monitoring studies to accurately assess the population dynamics under prolonged human pressures. Consequently, future studies should expand the temporal dimension of the research to acquire a more precise understanding of the survival pressures engendered by human influences on the Reeves’ turtle populations.

## Figures and Tables

**Table 1:** Ecological factors between selected and random quadrats in Reeves’ turtles in Qichun County, Hubei Province, China.

Habitat Type	Quadrat Size (m × m)	Factors	Mean ± SE		Paired *t*-Test or Wilcoxon Signed-Rank Test		
			Selected Habitat	Random Habitat	*t* or *Z*	*df*	*p*
Aquatic	10 × 10	Canopy cover (%)	3.04 ± 0.98	2.23 ± 0.99	−1.519	-	0.129
	10 × 10	Vegetation cover (%)	62.23 ± 4.33	50.00 ± 4.21	−3.430	-	0.001
	10 × 10	pH	7.16 ± 0.06	7.12 ± 0.06	1.182	73	0.241
	1 × 1	Canopy cover (%)	4.32 ± 1.60	4.12 ± 1.86	−0.315	-	0.753
	1 × 1	Shelter cover (%)	90.14 ± 2.14	43.18 ± 5.13	−6.032	-	<0.001
	1 × 1	Shelter height (cm)	50.81 ± 3.66	16.13 ± 3.00	−6.783	-	<0.001
	1 × 1	Depth of water (cm)	38.79 ± 3.83	43.65 ± 4.34	−2.009	-	0.045
	1 × 1	Distance from edge (m)	2.02 ± 0.23	4.00 ± 0.37	−4.288	-	<0.001
	1 × 1	Water flow velocity (cm/s)	0.12 ± 0.09	0.22 ± 0.16	−1.342		0.180
Terrestrial	10 × 10	Canopy cover (%)	23.33 ± 7.87	33.61 ± 10.16	−1.126	-	0.260
	10 × 10	Vegetation cover (%)	84.17 ± 7.46	74.72 ± 7.82	−1.521	-	0.128
	1 × 1	Canopy cover (%)	24.44 ± 8.82	46.67 ± 10.76	−2.100	-	0.036
	1 × 1	Slope gradient (°)	4.28 ± 2.15	4.56 ± 1.17	−1.540	-	0.124
	1 × 1	Herbage cover (%)	78.33 ± 8.98	55.83 ± 8.25	−2.347	-	0.019
	1 × 1	Herbage height (cm)	91.05 ± 19.57	58.84 ± 18.53	1.670	17	0.113
	1 × 1	Leaf litter cover (%)	27.22 ± 8.77	27.78 ± 10.15	−0.169	-	0.866
	1 × 1	Leaf litter thickness (cm)	2.18 ± 0.61	1.44 ± 0.47	−1.112	-	0.266
	1 × 1	Distance from water (m)	13.85 ± 3.90	13.08 ± 3.46	−0.237	-	0.813
	1 × 1	Distance from the field edge (m)	1.44 ± 0.55	3.90 ± 0.83	−2.724	-	0.006
	1 × 1	Dead grass cover (%)	64.44 ± 9.33	21.39 ± 8.30	−2.921	-	0.003
	1 × 1	Dead grass thickness (cm)	17.93 ± 2.77	5.98 ± 1.75	3.735	17	0.002

**Table 2:** Ecological factors comparison between females and males in Reeves’ turtles in Qichun County, Hubei Province, China.

Habitat Type	Quadrat Size (m × m)	Factors	Mean ± SE		Independent-Samples *t*-Test or Mann–Whitney U Test		
			Females	Males	*t* or *Z*	*df*	*p*
Aquatic	10 × 10	Canopy cover (%)	4.02 ± 1.50	1.43 ± 0.67	−0.882	-	0.378
	10 × 10	Distance from human settlements (m)	146.09 ± 10.17	155.86 ± 89.62	−0.495	46.364	0.623
	10 × 10	Distance from human disturbances (m)	40.31 ± 8.22	57.03 ± 12.06	−0.703	-	0.482
	10 × 10	Vegetation cover (%)	68.70 ± 5.15	51.61 ± 7.37	−1.947	-	0.051
	10 × 10	pH	7.03 ± 0.07	7.37 ± 0.08	−3.242	72	0.002
	1 × 1	Canopy cover (%)	5.43 ± 2.44	2.50 ± 1.32	−0.443	-	0.658
	1 × 1	Shelter cover (%)	91.30 ± 2.30	88.21 ± 4.24	−0.530	-	0.596
	1 × 1	Shelter height (cm)	51.50 ± 4.69	49.70 ± 5.95	0.237	72	0.814
	1 × 1	Depth of water (cm)	36.94 ± 4.98	41.83 ± 6.05	−0.725	-	0.469
	1 × 1	Distance from edge (m)	1.99 ± 0.27	2.06 ± 0.41	−0.152	72	0.879
	1 × 1	Water flow velocity (cm/s)	0.11 ± 0.11	0.14 ± 0.14	−0.337	-	0.736
Terrestrial	10 × 10	Canopy cover (%)	7.27 ± 4.83	48.57 ± 14.71	−3.165	16	0.006
	10 × 10	Distance from human settlements (m)	120.64 ± 21.29	183.57 ± 21.36	−1.982	16	0.065
	10 × 10	Distance from human disturbances (m)	26.12 ± 12.39	20.93 ± 4.23	0.323	16	0.751
	10 × 10	Vegetation cover (%)	90.00 ± 6.74	75.00 ± 16.22	−0.746	-	0.456
	1 × 1	Canopy cover (%)	3.18 ± 7.83	57.86 ± 15.73	−3.437	6.272	0.013
	1 × 1	Slope gradient (°)	2.09 ± 1.24	7.71 ± 5.15	−1.185	-	0.236
	1 × 1	Herbage cover (%)	86.36 ± 9.75	65.71 ± 17.16	−1.091	-	0.275
	1 × 1	Herbage height (cm)	98.77 ± 28.21	78.91 ± 25.81	0.483	16	0.635
	1 × 1	Leaf litter cover (%)	16.36 ± 5.92	44.29 ± 19.74	−1.355	7.093	0.217
	1 × 1	Leaf litter thickness (cm)	1.75 ± 0.69	2.86 ± 1.17	−0.871	16	0.396
	1 × 1	Distance from water (m)	14.36 ± 5.29	13.04 ± 6.10	0.160	16	0.875
	1 × 1	Distance from the field edge (m)	0.59 ± 0.23	2.77 ± 1.24	−1.724	6.430	0.132
	1 × 1	Dead grass cover (%)	71.82 ± 9.61	52.86 ± 18.86	0.896	9.151	0.393
	1 × 1	Dead grass thickness (cm)	20.02 ± 2.79	14.66 ± 5.72	0.940	16	0.361

**Table 3:** Stepwise discriminant analysis between selected and random habitats in Reeves’ turtles in Qichun County, Hubei Province, China.

Habitat Type	Factors	Discriminant Coefficients	Wilks’ *λ*	*F*	*p*
Aquatic	Shelter cover (%)	0.803	0.708	60.108	<0.001
	Shelter height (cm)	0.568	0.634	41.904	<0.001
	Distance from edge (m)	−0.596	0.553	38.789	<0.001
	Depth of water (cm)	0.781	0.485	38.021	<0.001
Terrestrial	Leaf litter thickness (cm)	0.817	0.719	13.298	0.001
	Dead grass thickness (cm)	1.164	0.573	12.287	<0.001

**Table 4:** Differences in line home range between male and female Reeves’ turtles in different seasons.

Season	Mean ± SE		Independent-Samples *t*-Test		
	Female	Male	*t*	*df*	*p*
Spring	282.46 ± 72.82	417.32 ± 164.02	−0.852	16	0.407
Summer	731.69 ± 134.78	193.90 ± 55.63	2.838	15	0.012
Autumn	299.95 ± 88.85	432.62 ± 172.88	−0.753	16	0.462
Winter	3.22 ± 1.69	5.33 ± 2.72	−0.697	16	0.496

## Data Availability

The data presented in this study are available in the Supplementary Materials.

## Supplementary Materials

The following supporting information can be downloaded at: https://www.mdpi.com/article/10.3390/ani13091514/s1, File S1: Hebitat selection; File S2: The home range.
